# Construction of Bio-Based Polyurethanes via Olefin Metathesis and Their Thermal Reversible Behavior

**DOI:** 10.3390/polym14173597

**Published:** 2022-08-31

**Authors:** Zizhao Liu, Gaosheng Gu, Junwu Chen, Zhongyu Duan, Binyuan Liu

**Affiliations:** Hebei Key Laboratory of Functional Polymer, School of Chemical Engineering and Technology, Hebei University of Technology, Tianjin 300401, China

**Keywords:** eugenol, bio-based polyurethane, olefin metathesis, thermal reversible

## Abstract

With the increase in awareness of environmental protection and the shortage of oil resources, bio-based polyurethane has attracted increasing attention due to its ecological friendliness, low cost and easy degradation. In this paper, using Eugenol (Eug) derived from plant essential oils as the raw resource, syringyl ethanol (Syol) was prepared, and three monomers were obtained by the reaction of the Eug or Syol with Hexamethylene diisocyanate (HDI)or 4,4′-methylene di (phenyl isocyanate) (MDI), respectively. Then, three novel bio-based polyurethanes, P(Eug-HDI), P(Syol-HDI) and P(Syol-MDI), were synthesized by olefin metathesis polymerization. The effects of the catalyst type, reaction solvent, reaction temperature, reaction time, molar ratio of catalyst dosage and metal salts on the Eug-HDI olefin metathesis polymerization were investigated in detail. Under the optimal conditions, the yield reached 64.7%. It is worth noting that the addition of metal Ni salts could significantly promote the polymerization, in which NiI_2_ could increase the yield to 86.6%. Furthermore, the thermal decomposition behaviors of these bio-based polyurethanes were explored by DSC and variable temperature infrared spectroscopy. The test results showed that P(Eug-HDI) had a reversible thermal decomposition and a certain self-healing performance. This paper provided a new method for the preparation of bio-based polyurethane.

## 1. Introduction

Polyurethane is a kind of polymer which is formed by a condensation reaction between hydroxyl (-OH) and isocyanate (NCO) to contain a urethane group (-NHCOO-) as a repeating unit. Due to its excellent toughness, wear resistance and adhesion, polyurethane has been widely used in coatings, adhesives, elastomers, synthetic leather, pavement materials and medical materials [1,2,3,4]. With the pursuit of green and sustainable polymer materials becoming an interesting topic, bio-based polyurethane has become the focus of attention [5]. Bio-based polyurethane is gradually replacing the traditional petroleum-based polyurethane with the advantages of ecological friendliness, causing little harm, its biodegradability, low price, and being renewable [6,7,8,9]. Eugenol, as a natural oil derived from plant essential oils such as clove oil, is a renewable chemical resource with a rich content and low cost in nature. The modification of hydroxyl and double bonds in eugenol can cause eugenol-based derivatives and polymers to possess the basic structure and similar properties of traditional petroleum-based polymers, showing a high potential for green chemistry.

The olefin metathesis reaction was found in the 1950s, and it has played an important role in organic synthesis so far. The double bonds that are usually chemically inert can interrupt the rearrangement and generate new carbon–carbon skeletons, which expands the imagination of new compounds and simplifies the synthesis steps. Olefin metathesis usually proceeds in mild reaction conditions, with a high yield, less byproducts and high stability, it is insensitive to water and oxygen has a strong functional group tolerance and most substrates do not require group protection [10].

**Scheme 1:**
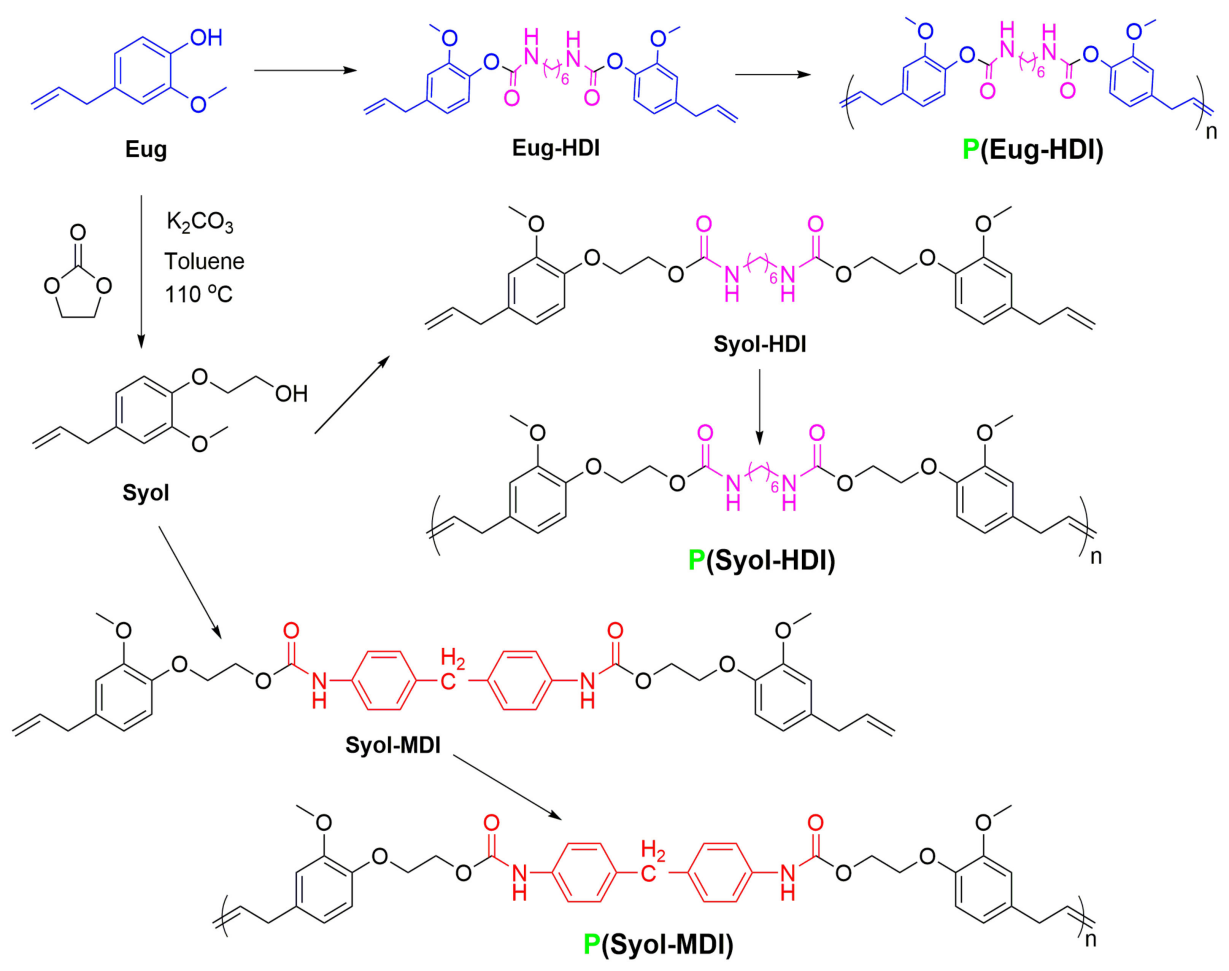
Synthesis diagram of monomers and polyurethanes.

Herein, different from the traditional polyol and isocyanate stepwise addition polymerization to provide polyurethane, Eug from natural plants was chosen as the raw material to prepare bio-based monomers with an amino ester bond and double bond at both ends by a reaction with diisocyanate. The bio-based monomers were polymerized by olefin metathesis reaction to obtain unsaturated polyurethane and the thermal decomposition or reversible behavior of three polyurethanes was investigated. The main content is shown in Scheme 1. The amino ester bond was introduced in the construction of the monomers, and the bio-based polyurethanes were prepared by olefin metathesis polymerization. The possibility of olefin metathesis polymerization of a biomass monomer was explored, which provided a new method for the preparation of bio-based polyurethane.

## 2. Materials and Methods

### 2.1. Materials

Eugenol (Eug, 99%), ethyl carbonate (EC, 99%), hexamethylene diisocyanate (HDI, 98%), 4,4′-methylene bis(isocyanate) (MDI, 98%), Grubbs catalyst I (G I, 98%), Grubbs catalyst II (G II, 98%), and Hoveyda-Grubbs II catalyst (H-G II, 98%) were purchased from Tianjin Keynes Biochemical Technology Co., Ltd. (Tianjin, China). Dibutyltin dilaurate (DBTDL, 98%) was purchased from the J & K Chemical Co., Ltd. (Beijing, China). The materials not mentioned were used as received.

### 2.2. Synthesis of Bio-Based Monomers

#### 2.2.1. Synthesis of Syringyl Ethanol (Syol)

Under argon protection, Eug (32.84 g, 0.2 mol), EC (26.42 g, 0.3 mol) and anhydrous potassium carbonate (4.14 g, 0.03 mol) in toluene (100 mL) were added into a 500 mL three-port bottle, dissolved and stirred, heated to 110 °C and refluxed for 16 h to stop the reaction. After cooling to room temperature, the crude product was extracted with ethyl acetate and water, dried with anhydrous sodium sulfate, then filtered and evaporated under reduced pressure. A column chromatography separation (petroleum ether:ethyl acetate = 2:1), rotary evaporation and vacuum drying for 12 h obtained a light yellow solid (36.06 g), and the yield was 86.6%. The structure of the Syol was characterized by FTIR and ^1^H NMR (Figures S1 and S7).

#### 2.2.2. Synthesis of Eugenol-Hexamethylene Diisocyanate (Eug-HDI) Monomer

Eug (32.84 g, 0.2 mol) and the catalyst DBTDL (0.63 g, 0.001 mol) in DMF (100 mL) were added to a 500 mL three-port bottle in an argon atmosphere, dissolved and stirred, heated to 85 °C, slowly dropped with HDI (16.82 g, 0.1 mol), refluxed for 3 h, then, the reaction was stopped. The mixture was cooled to room temperature with argon, recrystallized with petroleum ether and ethyl acetate (*v*:*v* = 1:1), then filtered and dried in a vacuum (60 °C) for 12 h to obtain white solid Eug-HDI (44.15 g), with a yield of 88.9%. The structure of the Eug-HDI was characterized by FTIR and ^1^H NMR (Figures S2 and S8).

#### 2.2.3. Synthesis of Syringyl Ethanol-Hexamethylene Diisocyanate (Syol-HDI) Monomer

Syol (41.64 g, 0.2 mol) and the catalyst DBTDL (0.63 g, 0.001 mol) in DMF (100 mL) were added into 500 mL three-necked bottles under an argon atmosphere, dissolved and stirred, heated to 85 °C, slowly dropped with HDI (16.82 g, 0.1 mol), refluxed for 3 h, then, the reaction was stopped. The white solid Syol-HDI (53.08 g) was obtained by the recrystallization of petroleum ether and ethyl acetate (*v*:*v* = 1:1) at room temperature with vacuum drying at 60 °C for 12 h after filtration. The yield of the product was 90.8%. The structure of the Syol-HDI was characterized by FTIR and ^1^H NMR (Figures S3 and S9).

#### 2.2.4. Synthesis of Syringyl Ethanol-4,4′-Methylenebis (Phenyl Isocyanate) (Syol-MDI) Monomer

Syol (41.64 g, 0.2 mol) and the catalyst DBTDL (0.63 g, 0.001 mol) in DMF (100 mL) were added into a 500 mL three-port bottle in an argon atmosphere, dissolved and stirred, and heated to 85 °C. Then, MDI (25.02 g, 0.1 mol) was dissolved in DMF, slowly dropped into a three-port bottle, and refluxed for 3 h to stop the reaction. The white solid Syol-MDI (53.46 g) was obtained by recrystallization with petroleum ether and ethyl acetate (*v*:*v* = 1:1) and vacuum drying at 60 °C for 12 h after filtration. The yield of the product was 80.2%. The structure of the Syol-MDI was characterized by FTIR and ^1^H NMR (Figures S3 and S10).

### 2.3. Polyurethane Prepared by Olefin Decomposition Polymerization

A certain amount of catalyst (0.2 mol%~4 mol%) and acyclic divinyl monomer (Eug-HDI (1.00 g, 2 mmol), or Syol-HDI (1.17 g, 2 mmol) or Syol-MDI (1.33 g, 2 mmol)), were weighed, added into a 25 mL reaction tube (glove box operation), and added with solvent in an argon atmosphere. The reaction was carried out in an oil bath at a preset temperature, and the argon was blowing in a reaction system. When the reaction was stopped, the reaction was quenched with vinyl ether, and a small amount of dichloromethane was dissolved. The mixture was placed into methanol and filtered. The filter cake was washed with methanol three times. The obtained solid crude product was dried in a vacuum drying oven for 16 h at 60 °C. Finally, the product was weighed and the yield was calculated.

### 2.4. Analysis and Measurements

Fourier transform infrared (FT-IR) spectra were recorded on a Bruker Vector-22 (Bruker, Karlsruhe, Germany) spectrometer with a resolution of 4 cm^−1^ in the range of 4000–400 cm^−1^, where the sample was dissolved in dichloromethane, applied to a KBr plate and then the solvents were removed. ^1^H NMR spectra were obtained on Bruker AVANCE-400 (Bruker, Karlsruhe, Germany) spectrometer at the scanning frequency of 400 MHz and a scanning range of 0–12 ppm with tetramethyl silane (TMS) as the internal standard. The molecular weight of the polymer was determined by gel permeation chromatography (GPC) on an Agilent PL-GPC 220 (Agilent Technologies, Waldbornn, Germany) at 35 °C and using THF as the mobile phase with the flow rate of 1 mL/min. An X-ray diffraction was measured on a MiniFlex600 (Rigaku Ltd., Tokyo, Japan.) within the regions of 8–60° at the scanning speed of 4°/min. A differential scanning calorimetry (DSC) was performed on a PE Diamond DSC (PerkinElmer, Waltham, MA, USA) by heating the samples from 60 to 200 °C at a rate of 10 °C/min under a N_2_ atmosphere. A thermogravimetric analysis (TGA) was carried out on a SDT-TG Q 600 (TA Instruments, Newcastle, WY, USA) by heating the samples from 20 to 800 °C in nitrogen at 10 °C/min.

## 3. Results and Discussion

### 3.1. Effect of Experimental Conditions on Polymerization Reaction

#### 3.1.1. Effect of Different Catalysts on Polymerization

The yields of olefin metathesis polymerization catalyzed by the catalysts, Grubbs I, Grubbs II and Hoveyda-Grubbs II are shown in Table 1, and the yields were 8.3%, 47.3% and 76.1%, respectively, indicating that the sequence of the catalytic activity was Hoveyda-Grubbs II > Grubbs II > Grubbs I. The difference in catalytic activity may be due to the poor thermal stability of the Grubbs catalyst, which was prone to decomposition at higher temperatures. However, the introduction of the Hoveyda-Grubbs II catalyst with a large volume of nucleophilic isopropyloxy chelating ligand improved the thermal stability; therefore, the Hoveyda-Grubbs II catalyst was selected for further exploration.

#### 3.1.2. Effect of a Different Solvent, Temperature and Time on Polymerization

**Figure 1:**
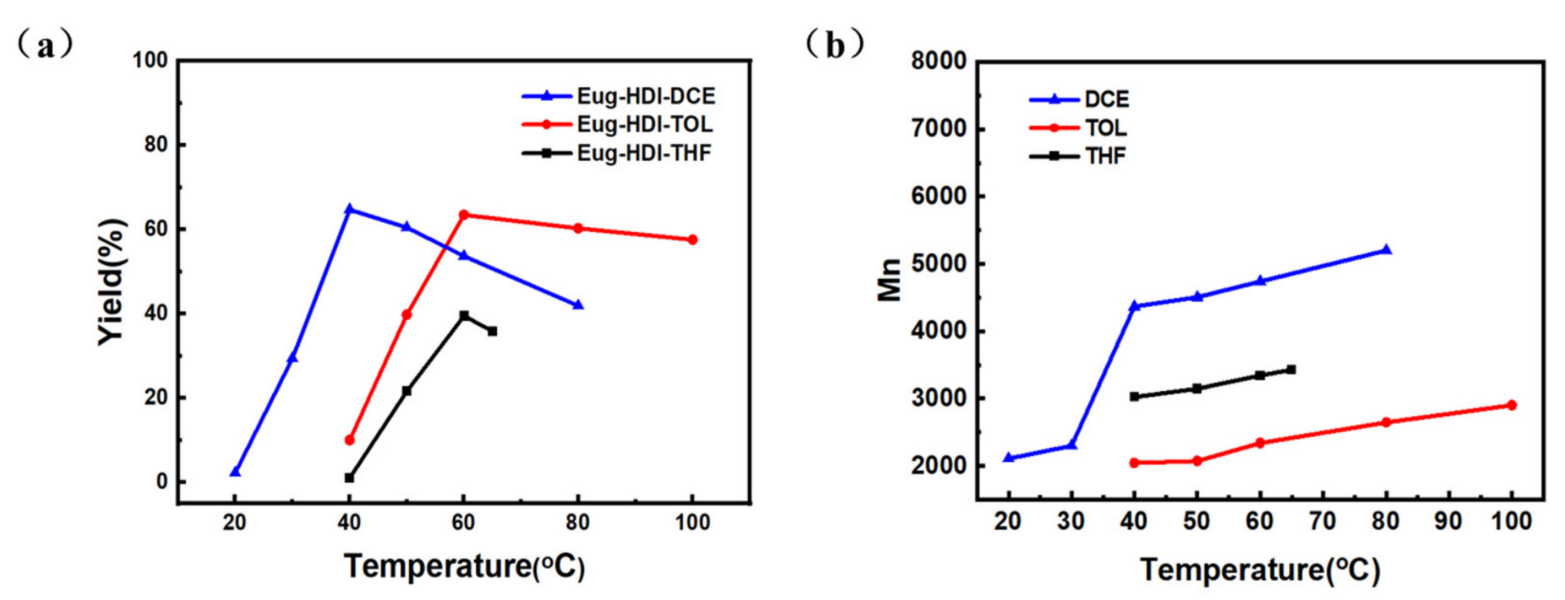
(**a**) Yield-temperature graph of Eug-HDI polymerization in different solvents, and (**b**) number average molecular weight-temperature graph of Eug-HDI polymerization in different solvents.

The yield-temperature diagram of the Eug-HDI polymerization in different solvents is shown in Figure 1a and entries 1–4, 5–9 and 10–15 in Table S1. The number average molecular weight-temperature diagram of the Eug-HDI polymerization in different solvents is shown in Figure 1b. It can be seen from the figure that dichloroethane (DCE) as a solvent is better than toluene (TOL) and tetrahydrofuran (THF), which may be due to the good solubility of DCE for monomers and polymers [11].

Among them, the reaction yield reached the highest (64.7%) at 40 °C with dichloroethane as the solvent (Figure 1a). After a comprehensive consideration, the optimum reaction temperature was 40 °C, because the yield and molecular weight were high. If you wanted to further improve the molecular weight by heating (Figure 1b), the yield would decrease sharply (Figure 1a). Since olefin metathesis is a reversible reaction, the product contains all possible combinations of olefins, and the proportion depends on the thermodynamic stability of each olefin [12,13]. Before 40 °C, the chain growth of the polymer was mainly dominated by the growth of a monomer to remove the ethylene, and an increasing temperature was conducive to the positive polycondensation. At this time, the yield and molecular weight were positively correlated with the temperature. After 40 °C, the chain growth of the polymer mainly consisted of two long chain complex decompositions to form a longer chain and a short chain (the major was a dimer). The dimer in the reaction system could not be precipitated in methanol due to its low molecular weight. The above methanol phase was collected, and a small amount of white powder was obtained after the solvent was removed by rotary evaporation. The NMR test was characterized as a mixture of mainly dimers (Figure S14).

**Figure 2:**
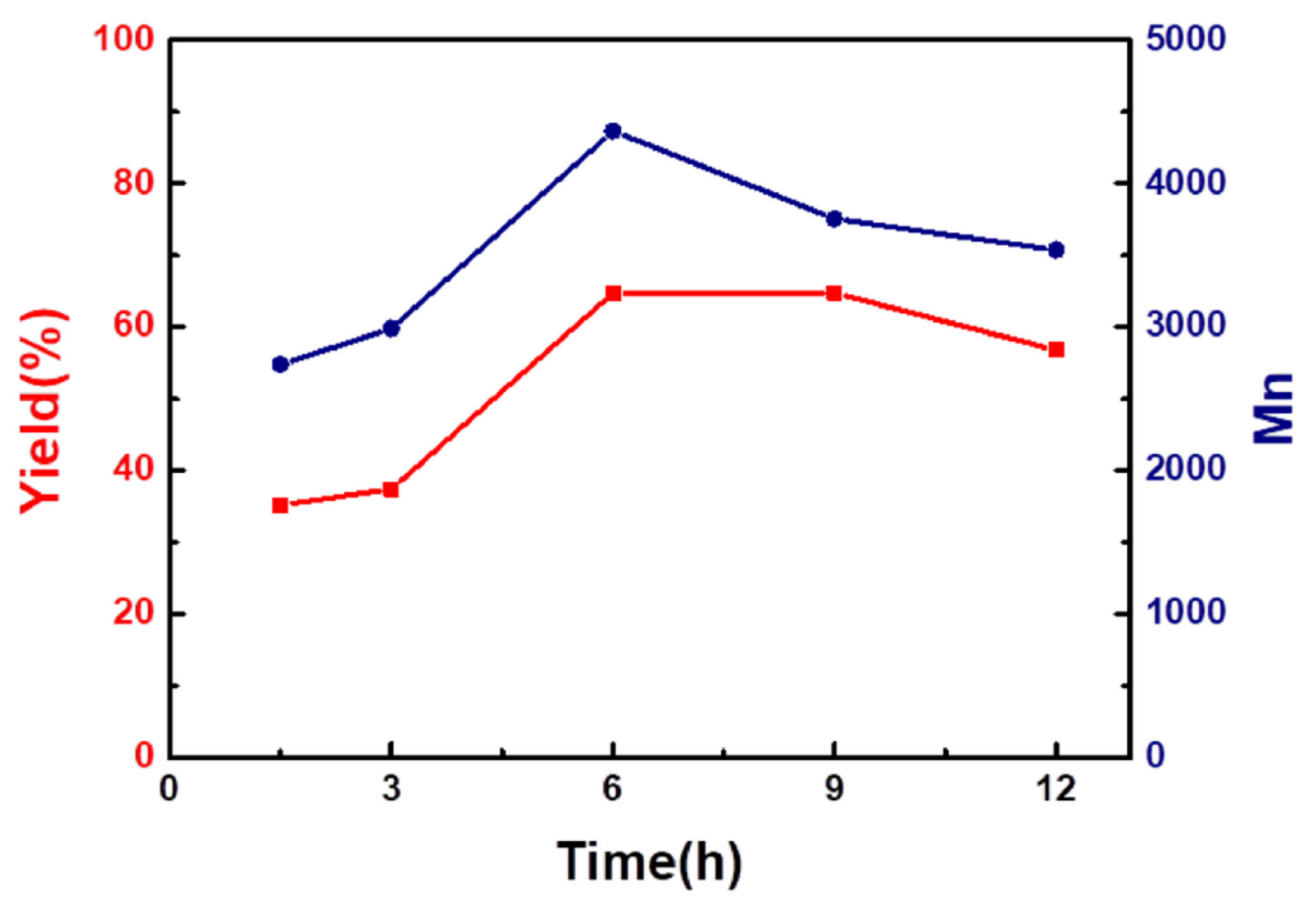
Yield (left) and number average molecular weight (right) vs. time graph of Eug-HDI polymerization.

The time gradient experiment of the Eug-HDI olefin metathesis polymerization was carried out at 40 °C in DCE. As shown in Figure 2 and entries 16–20 in Table S1, with the extension of the reaction time, the polymerization yield and molecular weight increased first and then decreased. When the reaction time was 6 h, the yield and molecular weight were the largest, indicating that 6 h was the best reaction time. Due to the reversible decomposition of olefins, the polycondensation reaction proceeded forward before 6 h, and the yield and molecular weight of the polymer increased with time. After 6 h, the reaction reached a balance and the chain no longer grew; after 6 h, with the extension of the reaction time, the molecular chain of the polymer transferred to a monomer or dimer, resulting in the decrease in molecular weight and yield.

#### 3.1.3. Effect of Catalyst Mole Ratio on the Polymerization Reaction

**Figure 3:**
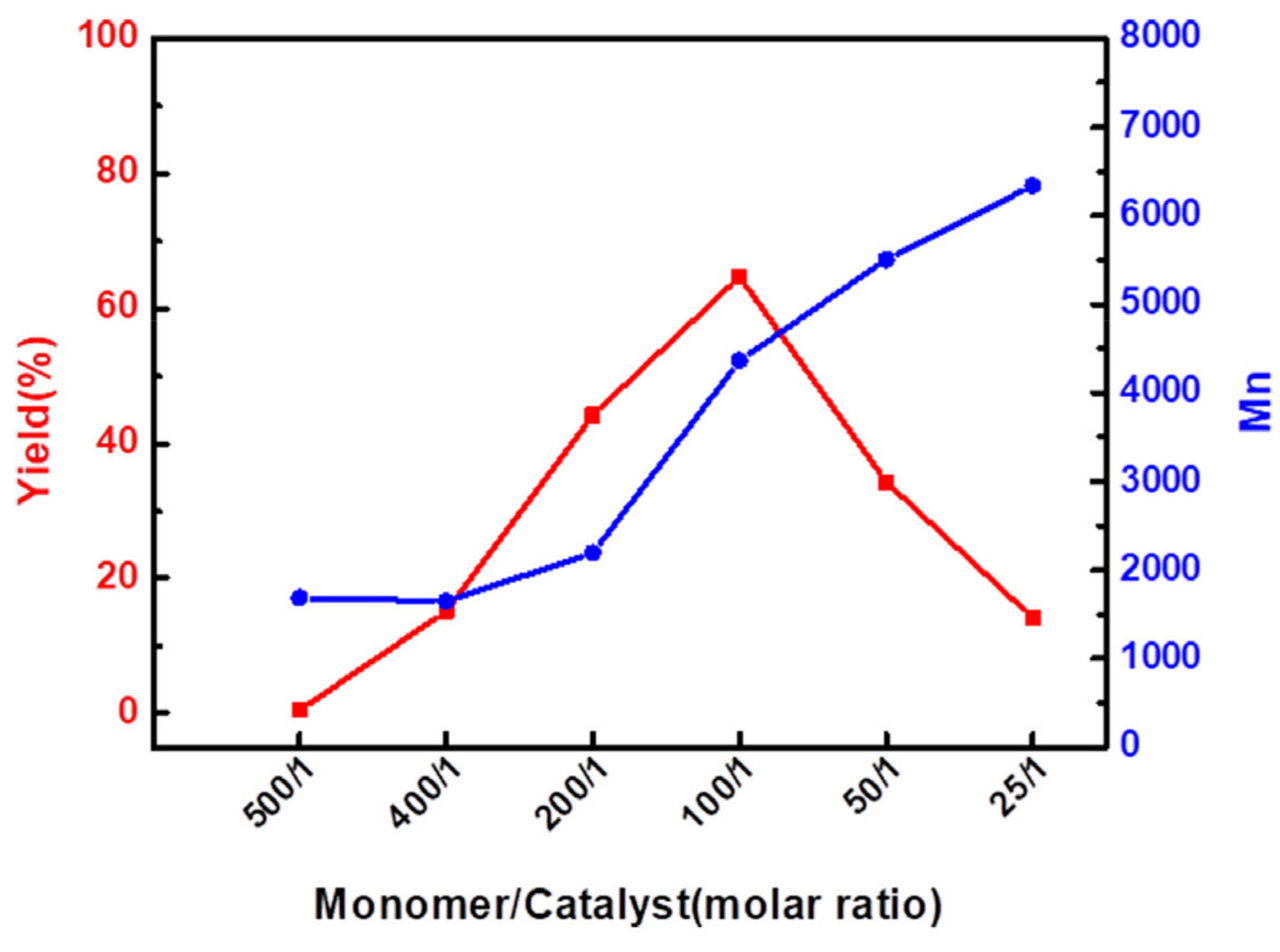
Yield (left) and number average molecular weight (right) vs. catalyst molar ratio of Eug-HDI polymerization.

The effects of the mole ratio of the *n* (monomer)/*n* (catalyst) on the olefin metathesis polymerization of Eug-HDI were investigated at 40 °C for 6 h with DCE as the solvent. The results in Figure 3 and Table S2 show that the mole ratio of *n* (monomer)/*n* (catalyst) = 100/1 was the best catalytic condition. At this condition, the catalyst reached saturation. Then, with a continued increase in the catalyst molar ratio, the yield decreased (Figure 3 (red line)) and a large number of dimers failed to precipitate in the methanol phase. A reasonable speculation on these experimental results and phenomena may be that there is a competitive relationship between too many catalytic active centers, so that a very small number of active centers grow into high molecular weight long chains, while most of them only form dimers.

#### 3.1.4. Effect of Metal Salts Addition on the Polymerization Reaction

**Figure 4:**
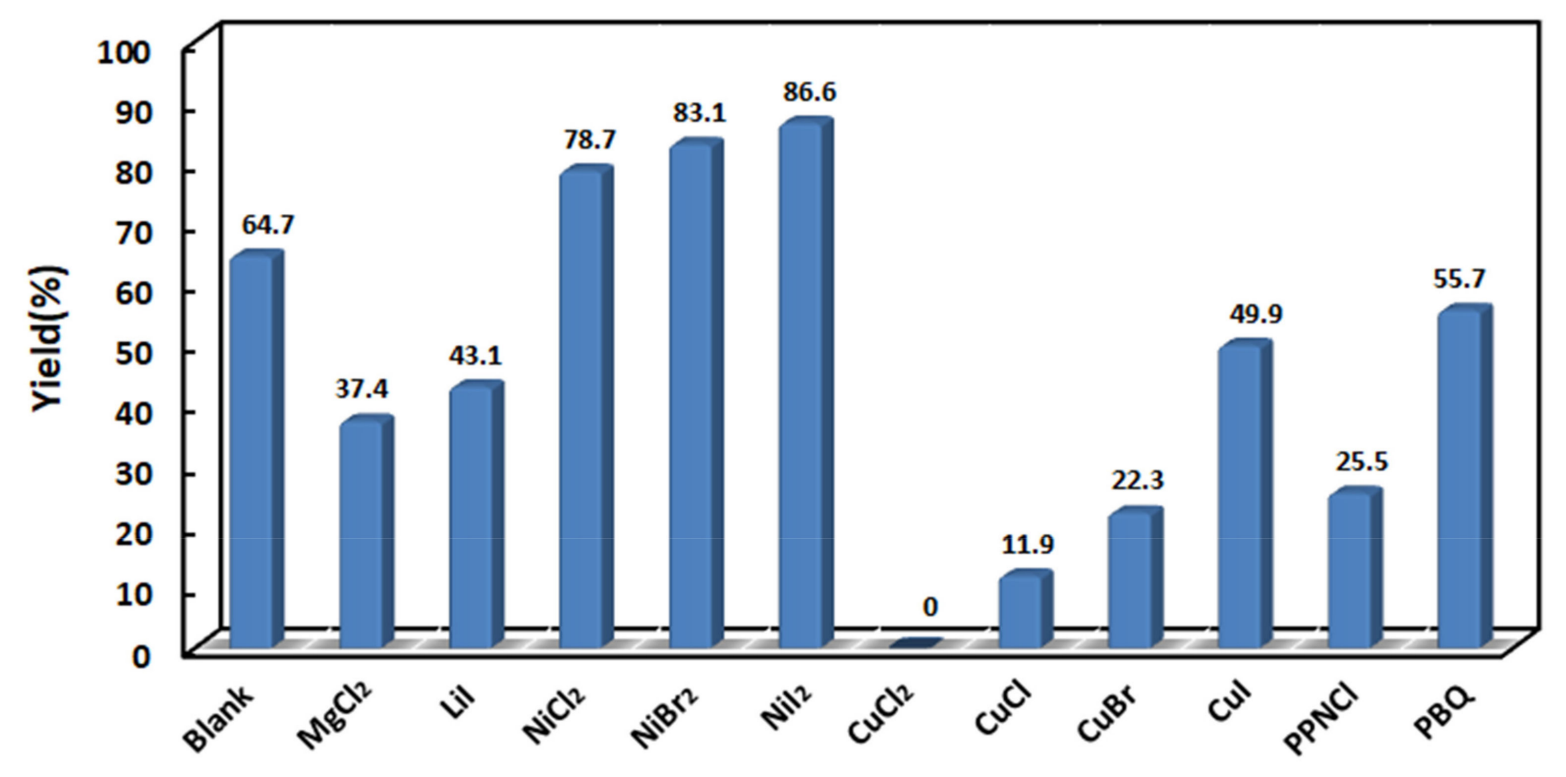
Yield vs. different metal salts of Eug-HDI polymerization.

Olefin metathesis refers to the process of carbon–carbon double bond breaking and recombining between molecules catalyzed by metal carbene. The addition of metal salts may make the metal carbene more stable, thereby increasing the stability of the catalyst and the yield in the reaction [14]. The effect of metal salts on Eug-HDI olefin metathesis polymerization was explored by adding different metal salts, with dichloroethane as the solvent, via a reaction at 40 °C for 6 h. As shown in Figure 4 and Table S3, most of the added metal salts decreased the yield, but the addition of Ni salt significantly increased the yield, indicating that Ni salt would make the metal carbene more stable, thereby increasing the catalytic activity. The nucleophilicity of halogen ions was as follows: I^−^ > Br^−^ > Cl^−^. The effect of different Ni salts on enhancing the catalytic activity was as follows: NiI_2_ > NiBr_2_ > NiCl_2_, which was consistent with the anionic nucleophilicity. The results showed that the stronger the nucleophilicity of the added metal salt anions, the higher the catalytic activity of the catalyst and the higher the polymerization yield. In contrast to nickel salt, the addition of cuprous salts reduces the catalytic activity and it plays a toxic role in the catalytic activity of olefin metathesis polymerization.

### 3.2. Characterization and Analysis of Polymerization Products

The infrared spectra of the Eug-HDI, Syol-HDI, Syol-MDI and their corresponding polymerization products are shown in Figures S4–S6. The NMR spectra of the P(Eug-HDI), P(Syol-HDI) and P(Syol-MDI) are shown in Figures S11–S13. The corresponding analysis is displayed in the Supplementary Materials.

#### 3.2.1. GPC Characterization

**Figure 5:**
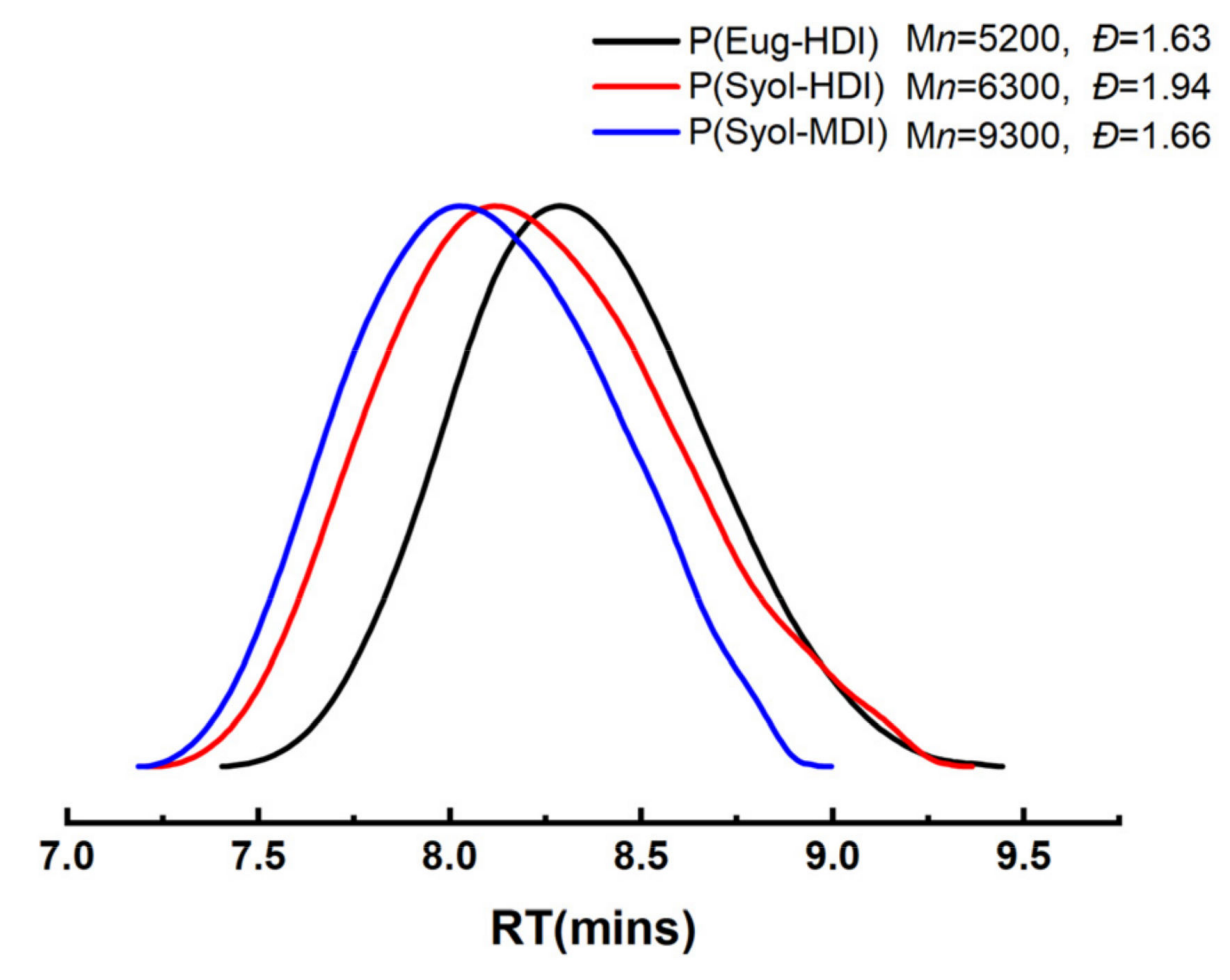
GPC curves of polymerization products.

The molecular weight distributions of the polymers are shown in Figure 5. P(Eug-HDI): a single peak, molecular weight of 5200 g/mol, and dispersion index of 1.63; P(Syol-HDI): a single peak, molecular weight of 6300 g/mol, and dispersion index of 1.94; P(Syol-MDI): a single peak, molecular weight of 9300 g/mol, and dispersion index of 1.66.

#### 3.2.2. Thermal Stability

**Figure 6:**
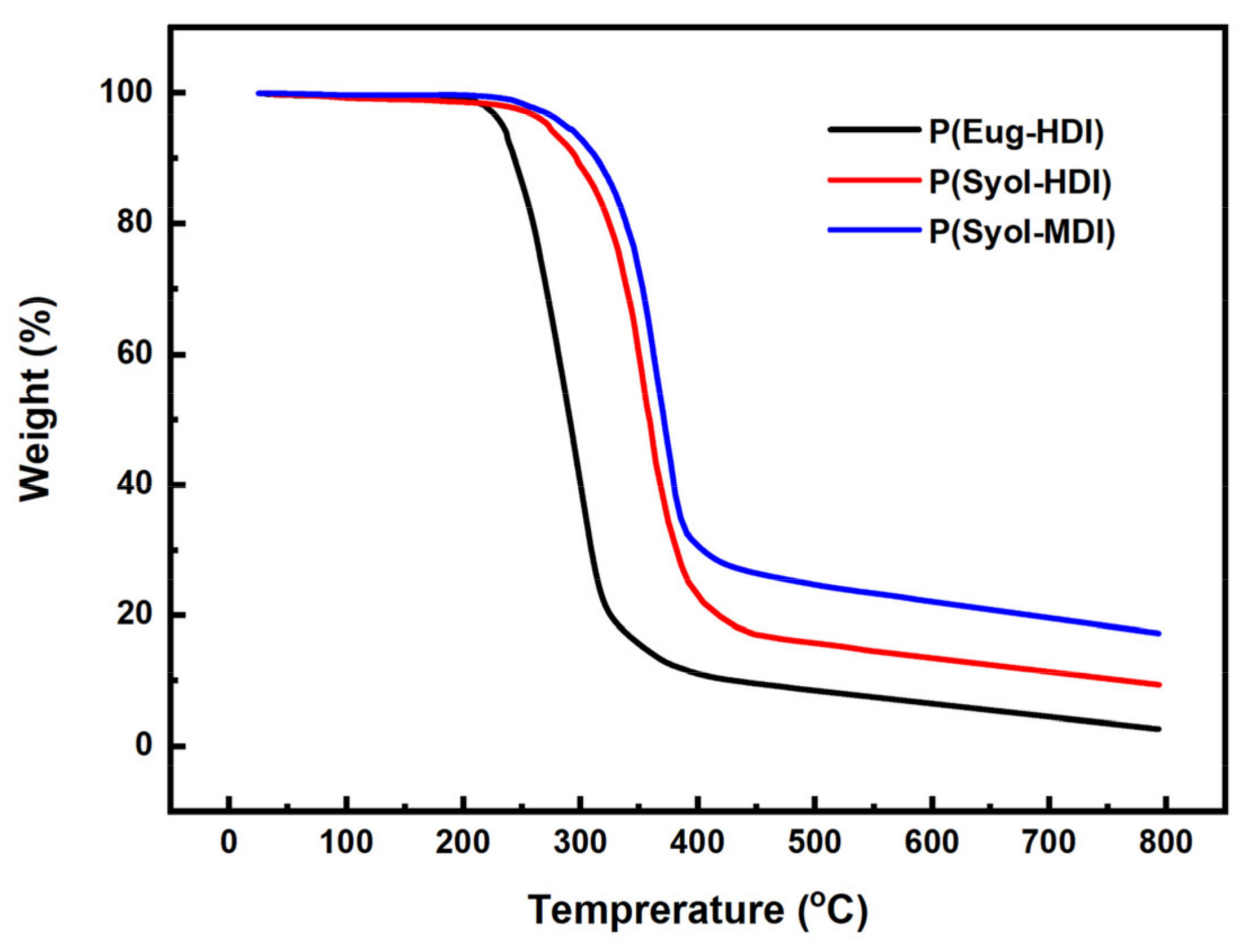
Thermogravimetric curve of different monomer polymerization products.

The thermogravimetric curves of the polymers are shown in Figure 6, where the P(Eug-HDI) began to degrade at 210 °C, and the loss was 5% at 233 °C and 10% at 243 °C, the P(Syol-HDI) began to degrade at 240 °C, with a loss of 5% at 273 °C and a loss of 10% at 296 °C, and the P(Syol-MDI) began to degrade at 245 °C, with a loss of 5% at 287 °C and a loss of 10% at 313 °C. It can be seen that the thermal stability of the three polymers was: P(Syol-MDI) ≥ P(Syol-HDI) > P(Eug-HDI).

The polymer decomposition temperature refers to the temperature when the macromolecular chain begins to break down or some groups decompose from the macromolecule when the polymer is heated. The thermal decomposition temperature of the polymer is related to the chemical structure (basic structural unit) and chain structure of the polymer. The chain structure includes the molecular weight, molecular weight distribution and branching degree. For polymers with the same structural unit, the higher the molecular weight of the polymer, the higher the thermal decomposition temperature is [15,16]. This phenomenon has been widely reported. In addition, due to the difficulty in achieving ‘controllable polymerization’ in the catalytic system, this paper did not prepare the polymers with different molecular weights in the same structural series; therefore, the influence of the molecular weight on the thermal decomposition is no longer discussed. The thermal decomposition temperature of polymers is closely related to factors such as their chemical structure [17]. Herein, the influence of different chemical structures on the thermal decomposition is mainly discussed and the thermal stability, namely, ester, ether > urea, carbamate > urea formic acid ester, biuret [18]. The chemical structure of the P(Syol-HDI) was two methylenes and ether bonds more than that of the P(Eug-HDI); therefore, the thermal stability of the P(Syol-HDI) was higher than that of the P(Eug-HDI). Compared with the P(Eug-HDI) and P(Syol-HDI) that contained hexamethylene, the P(Syol-MDI), that contained a benzene ring rigid structure, the chain segment was hard and the thermal stability was stronger [19]; therefore, the thermal stability of the three polymers was consistent with their chemical structure. The thermal stability of the polymers prepared in this paper was similar to that of the polymers prepared by the traditional route, and most of them began to decompose at 200–300 °C [20].

### 3.3. Study on Thermal Decomposition and Reversible Behavior

The amino ester bond formed by a hydroxyl (-OH) and isocyanate group (NCO) will be thermally decomposed to produce new NCO groups. Whether it can react with hydroxyl to form an amino ester bond after cooling is the key to reversible behavior [21,22,23]. The difficulty of thermal decomposition or thermal reversible behavior is related to the structure of isocyanate, the type of hydroxyl and the reaction conditions.

The most important research of thermal decomposition or thermal reversible behavior is the study of the decomposition temperature. The commonly used research methods are FTIR [24,25], DSC [26,27] and TGA [28,29]. If a heating device is added to the infrared test process to test the infrared spectra at different temperatures and at different times, the thermal decomposition or thermal reversible behavior can also be studied [30,31,32].

#### 3.3.1. XRD Characterization

**Figure 7:**
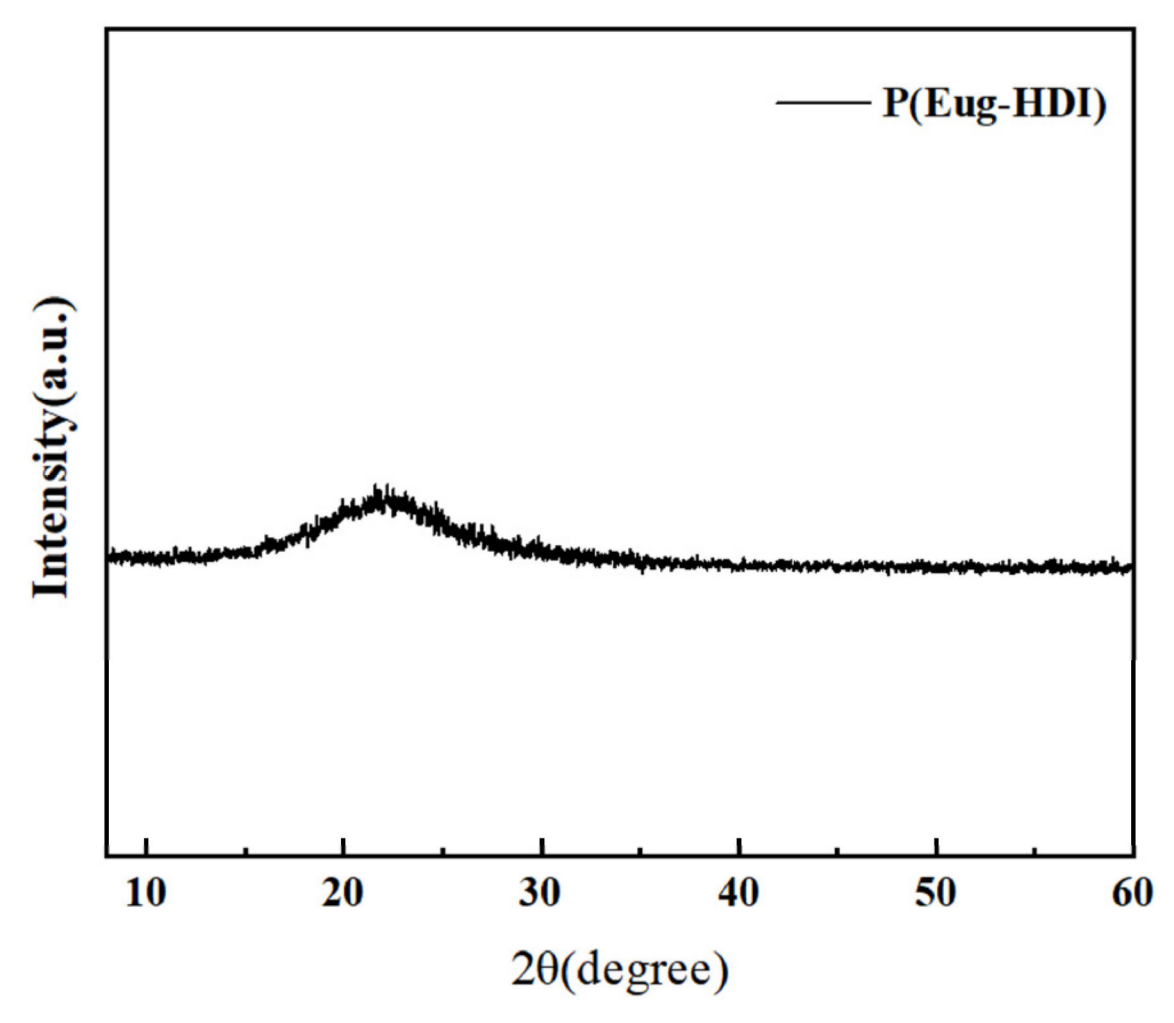
X-ray diffraction spectrum of P(Eug-HDI).

X-ray diffraction (XRD) can be used to determine whether a polymer is crystalline [33]. There are obvious sharp diffraction peaks in the diffraction spectrum, which are crystalline materials and the sharper the peak is, the more perfect the crystallization is. If there is only a wide dispersion peak, it is an amorphous material. The XRD curve of the polymer is shown in Figure 7. There was only a broad diffraction peak in the spectrum, indicating that the polymer was amorphous.

#### 3.3.2. Studies on Thermal Decomposition Behavior by DSC

**Figure 8:**
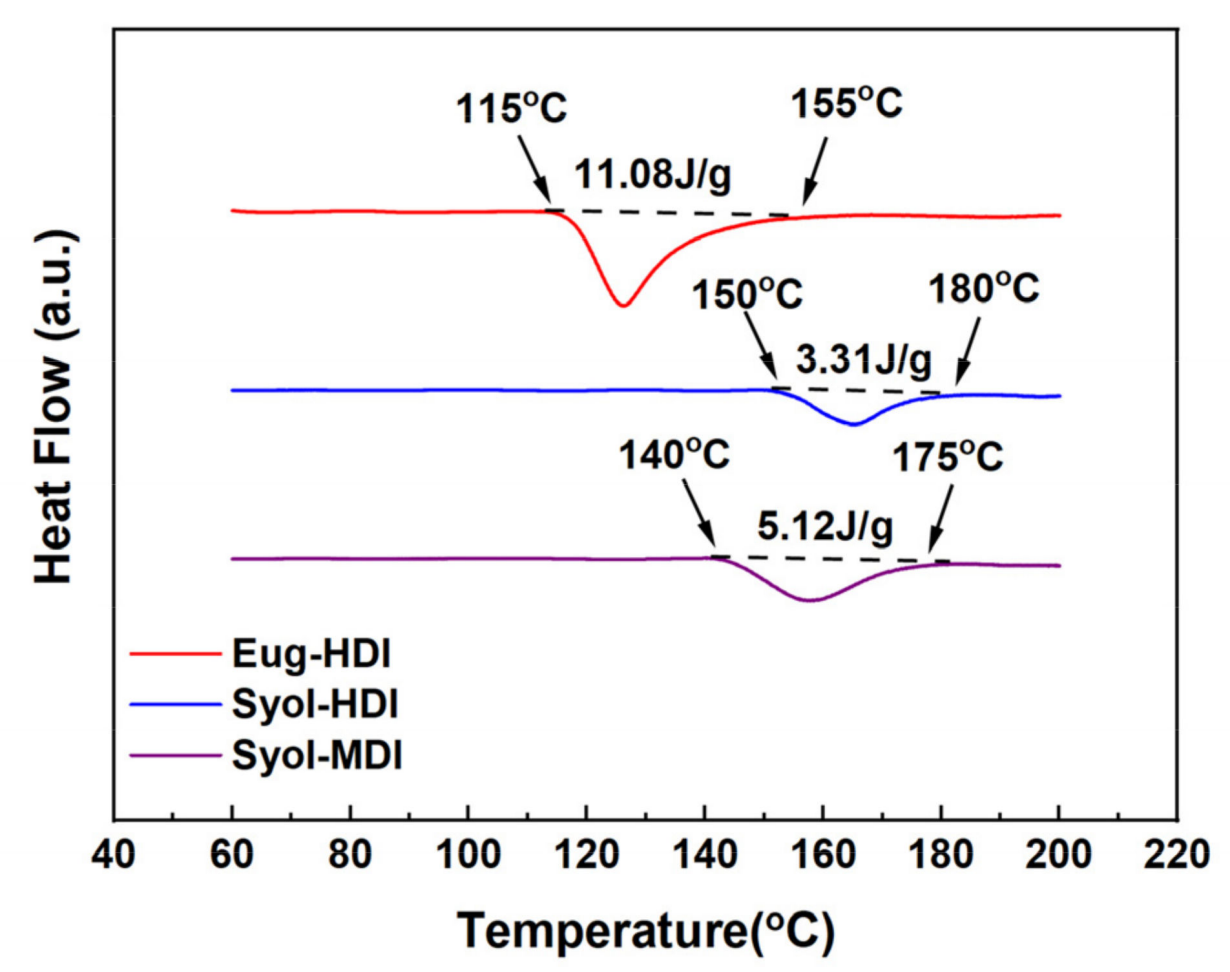
DSC curves of polymers.

A thermal decomposition reaction is an endothermic reaction, and the XRD characterization shows that the polymer was amorphous; therefore, the difficulty of thermal decomposition can be reflected by the size of the endothermic peak on the DSC curve and its initial temperature. The DSC curves of the polymerization products of different carbamate monomers are shown in Figure 8. At the heating rate of 10 °C/min, from 60 °C to 200 °C, the heat absorbed by the polymer P(Eug-HDI) was 11.08 J/g, and the heat absorbed by the P(Syol-HDI) and P(Syol-MDI) was 3.31 J/g and 5.12 J/g, respectively. It can be seen that the heat absorbed by the polymer P(Eug-HDI) was the largest and the endothermic peak was also the most obvious. Compared with the DSC curves of the polymerization products, it can also be observed that the endothermic peak of the P(Eug-HDI) appeared at 120 °C, indicating that the thermal decomposition had occurred at this temperature. The initial thermal decomposition temperature of the P(Syol-HDI) was 150 °C, and the initial thermal decomposition temperature of the P(Syol-MDI) was 140 °C. The above results show that P(Eug-HDI) is prone to a thermal decomposition reaction and has a greater degree of thermal decomposition at the same temperature than the others.

#### 3.3.3. Studies on Thermal Decomposition or Thermal Reversible Behavior by Infrared

**Figure 9:**
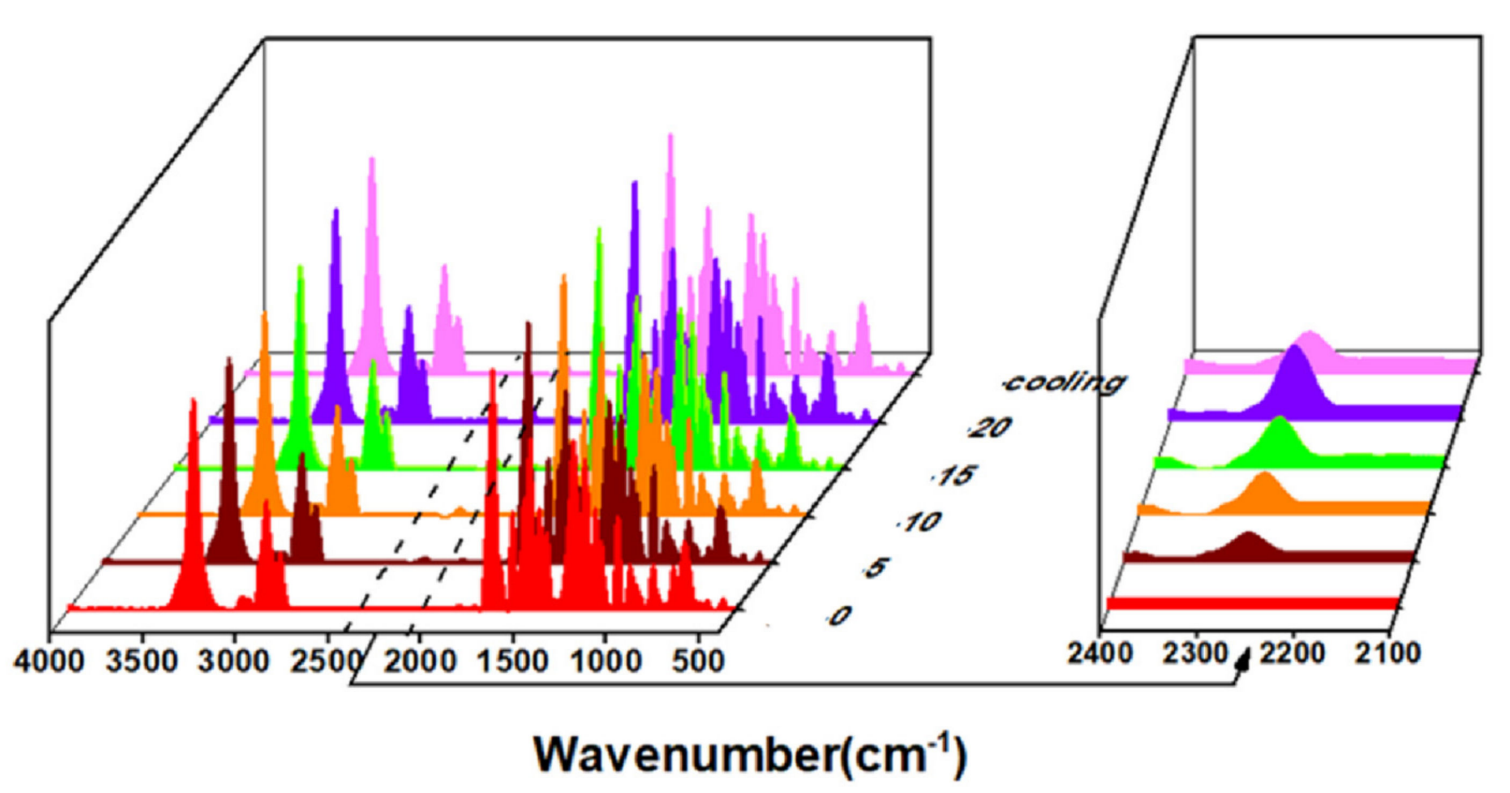
The IR spectra of P(Eug-HDI) vs. different heating time.

The thermal decomposition occurs on the isocyanate (NCO) group and the amino ester bond formed by the hydroxyl group, which produces a free NCO group; therefore, the change of the NCO peak during heating can be measured by infrared spectroscopy to reflect the thermal decomposition behavior. If the NCO group produced by thermal decomposition can be ammoniated again, it is a thermal reversible behavior. The P(Eug-HDI) was heated at 120 °C for 20 min, and the infrared spectra of 0 min (before heating), 5 min, 10 min, 15 min, 20 min and 10 min after cooling were measured, respectively. The results are shown in Figure 9. It can be qualitatively seen that with the increase in heating time, the characteristic absorption peak of the isocyanate near 2250 cm^−1^ appeared and the peak intensity gradually increased, indicating that the polymer P(Eug-HDI) had begun thermal decomposition at this temperature. On the contrary, after cooling for 10 min, the characteristic absorption peak of the isocyanate near 2250 cm^−1^ weakened. This was because there was a dynamic equilibrium between the thermal decomposition and re-urethane reaction. Increasing the temperature will cause the equilibrium to move in the direction of endothermic thermal decomposition, and reducing the temperature will cause the equilibrium to move in the direction of exothermic re-ammonia esterification. The above results indicate that the phenolic hydroxyl polyurethane P(Eug-HDI) exhibited the thermal reversible behavior of thermal decomposition by heating and the re-urethane reaction by cooling. This reveals that the P(Eug-HDI) had a certain self-healing property.

**Figure 10:**
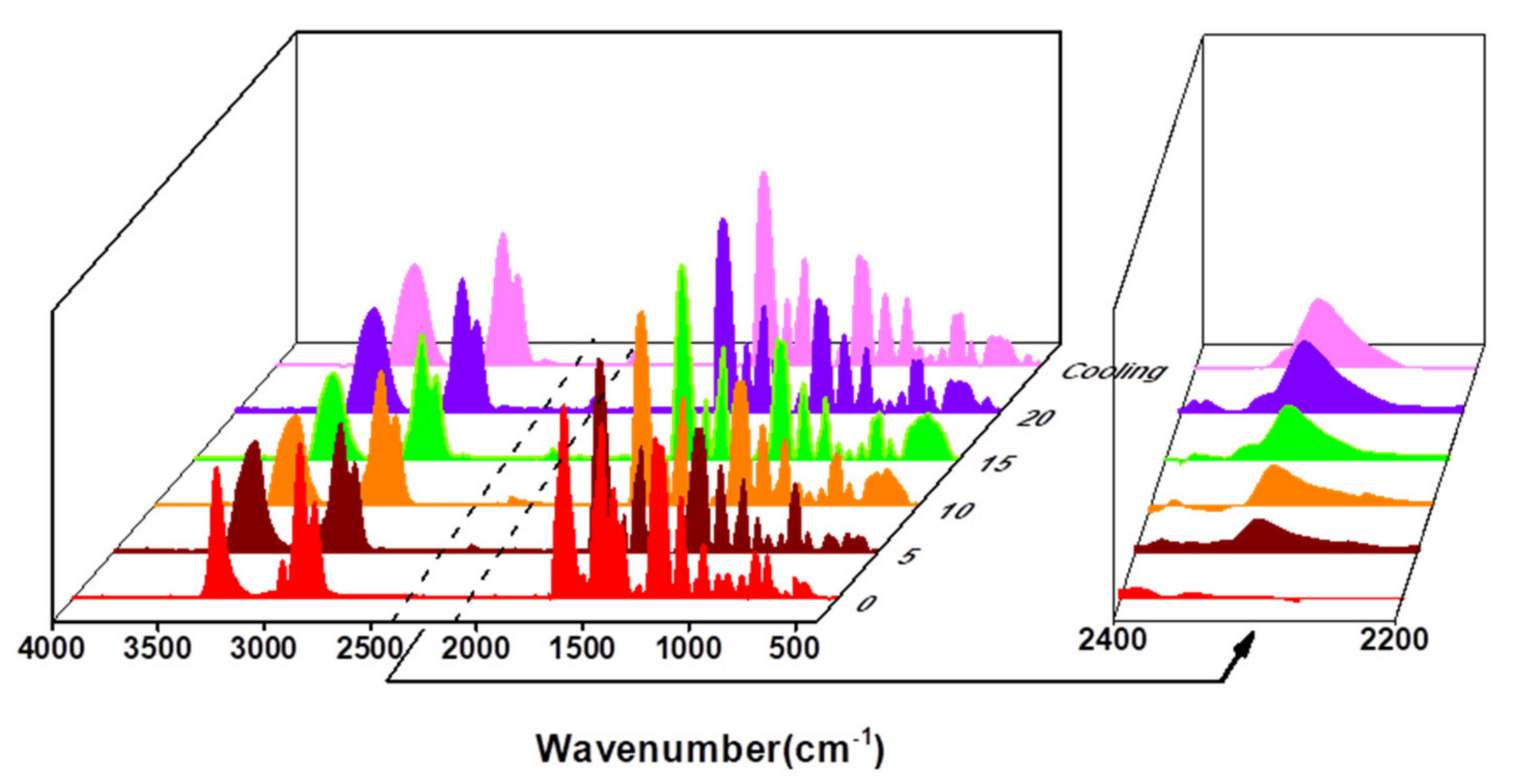
The IR spectra of P(Syol-HDI) vs. different heating time.

**Figure 11:**
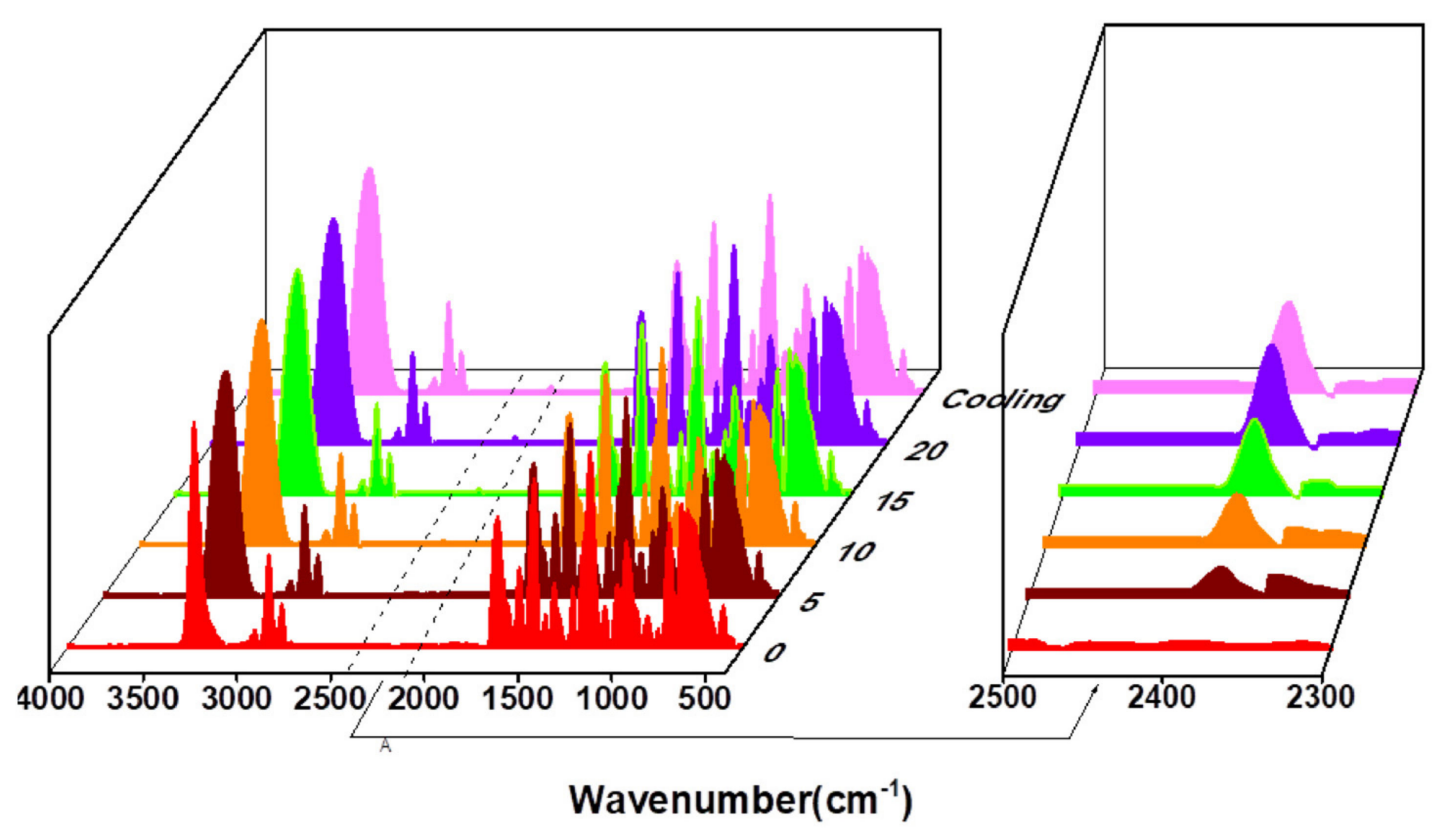
The IR spectra of P(Syol-MDI) vs. different heating time.

Similarly, the P(Syol-HDI) and P(Syol-MDI) were heated at 150 °C and 140 °C for 20 min, respectively. The infrared spectra of 0 min (before heating), 5 min, 10 min, 15 min, 20 min and 10 min after cooling were measured, respectively. The results are shown in Figure 10 and Figure 11. It can be qualitatively seen that in the heating process, the stretching vibration peak of N-H on the amino ester bond at 3320 cm^−1^ was widened and moved to the high field to the stretching vibration peak of the hydroxyl (OH) at 3350 cm^−1^, indicating that part of the amino ester bond in the polymer was thermally decomposed and hydroxyl was regenerated. The characteristic absorption peak of the isocyanate appeared near 2300 cm^−1^, and the peak intensity increased with the increased heating time, but the characteristic absorption peak of the NCO did not change after cooling for 10 min. The above results indicate that the alcohol hydroxyl polyurethanes P(Syol-HDI) and P(Syol-MDI) had only thermal decomposition behaviors, they could not cause a re-urethane reaction and that this was irreversible.

## 4. Conclusions

In summary, Syol was prepared by the ring-opening substitution method using Eug as the raw material. Eug and Syol reacted with diisocyanate, respectively, to synthesize biological monomers (namely, Eug-HDI, Syol-HDI and Syol-MDI), then, the bio-based polyurethanes P(Eug-HDI), P(Syol-HDI) and P(Syol-MDI) were successfully prepared via olefin metathesis polymerization. The effects of the catalyst type, solvent, reaction temperature, time and catalyst mole ratio, and metal salts on the polymerization were investigated. The addition of metal Ni salts significantly promoted polymerization, in which NiI_2_ increased the yield to 86.6%. The structures and properties of the polymers were characterized and analyzed by FTIR, ^1^H NMR, GPC, and XRD tests, and the thermal decomposition or thermal reversible behaviors were explored by DSC and variable temperature infrared spectroscopy. The test results showed that P(Eug-HDI) had a reversible thermal decomposition and certain self-healing performance. Furthermore, the Eugenol selected in the paper comes from nature and has an abundant yield and renewability. The paper has provided a new idea for the preparation of bio-based polyurethane.

## Figures and Tables

**Table 1:** Eug-HDI olefin metathesis under different catalysts.

Entry	Eug-HDI/Cat. (Molar Ratio)	Cat.	Solvent (mL)	T (°C)	t (h)	Conv. (%)
1	25/1	Grubbs I	TOL	80	6	8.3
2	25/1	Grubbs II	TOL	80	6	47.3
3	25/1	Hoveyda-Grubbs II	TOL	80	6	76.1

## Data Availability

The data presented in this study are available.

## Supplementary Materials

The following supporting information can be downloaded at: https://www.mdpi.com/article/10.3390/polym14173597/s1, Figure S1: IR spectra of Eug and Syol. Figure S2: IR spectra of Eug and Eug-HDI. Figure S3: IR spectra of Syol, Syol-HDI and Syol-MDI. Figures S4–S6: The IR spectra of Eug-HDI, Syol-HDI, Syol-MDI and their corresponding polymers. Figures S7–S10: The ^1^H NMR spectra of Syol, Eug-HDI, Syol-HDI, Syol-MDI, respectively. Figures S11–S13: The ^1^H NMR spectra of P(Eug-HDI), P(Syol-HDI) and P(Syol-MDI). Figure S14: The 1H NMR spectrum of product in methanol filtrateby dried by rotary evaporation. Table S1: Olefin metathesis polymerization of Eug-HDI under different solvents, temperatures and reaction times. Table S2: Olefin metathesis polymerization of Eug-HDI under different catalyst molar ratios. Table S3: Olefin metathesis polymerization of Eug-HDI with different metal salts.
