# Supplementary material for: Construction of Bio-Based Polyurethanes via Olefin Metathesis and Their Thermal Reversible Behavior
Source: Polymers (Basel). 2022 Aug 31;14(17):3597. doi: 10.3390/polym14173597 (PMC9460269; doi:10.3390/polym14173597)
Supplement: Supplementary file 1 [file polymers-14-03597-s001.zip › polymers-1852790-supplementary.pdf]

polymers

Supplementary Materials

Construction of Bio-based Polyurethane via Olefin Metathesis and Their Thermal Reversible Behavior

Zizhao Liu, Gaosheng Gu, Junwu Chen, Zhongyu Duan*, and Binyuan Liu*

[1] Hebei Key Laboratory of Functional Polymer, School of Chemical Engineering and Technology, Hebei University of Technology, Tianjin, China
* Correspondence: zyduan@hebut.edu.cn；byliu@hebut.edu.cn

Citation: Liu, Z.; Gu, G.; Chen, J.; Duan, Z.; Liu, B. Construction of Bio-Based Polyurethanes via Olefin Metathesis and Their Thermal Reversible Behavior. *Polymers* **2022**, *14*, 3597. https://doi.org/10.3390/polym14173597

Academic Editor: Alexey Iordanskii

Received: 21 July 2022
Accepted: 26 August 2022
Published: 31 August 2022

Publisher's Note: MDPI stays neutral with regard to jurisdictional claims in published maps and institutional affiliations.

Copyright: © 2022 by the authors. Submitted for possible open access publication under the terms and conditions of the Creative Commons Attribution (CC BY) license (https://creativecommons.org/licenses/by/4.0/).

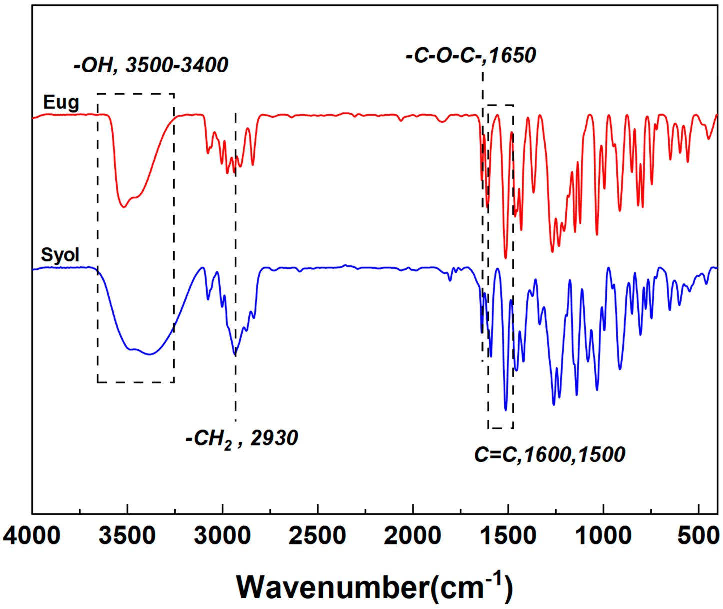

Figure S1. IR spectra of Eug and Syol.

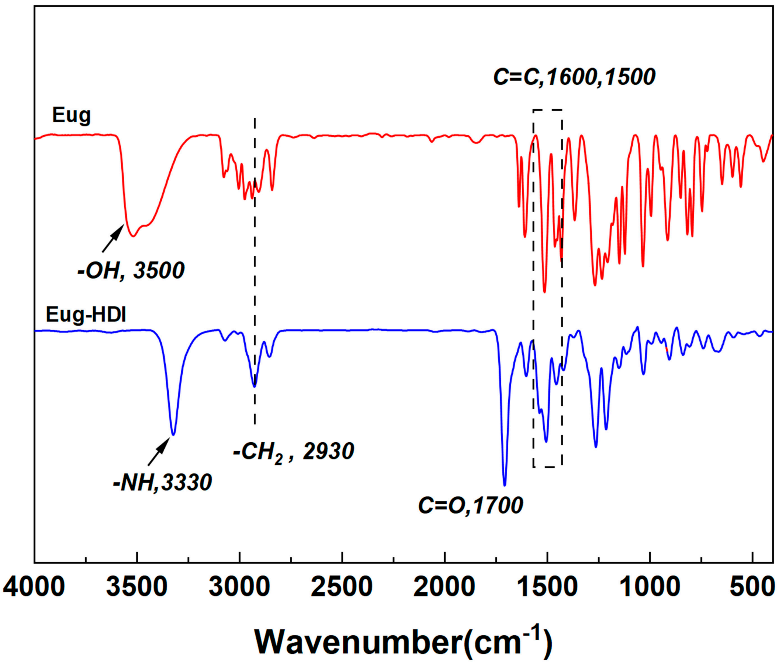

Figure S2. IR spectra of Eug and Eug-HDI.

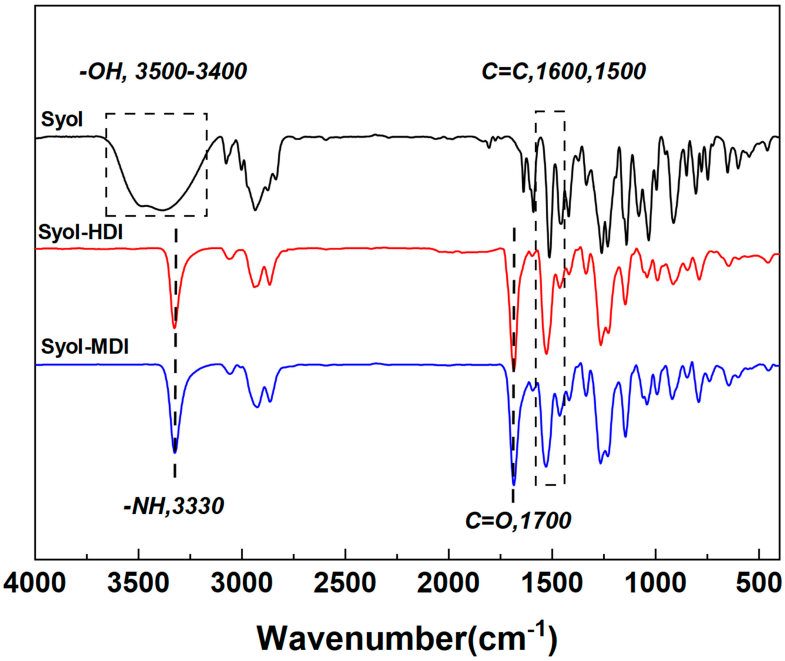

Figure S3. IR spectra of Syol, Syol-HDI and Syol-MDI.

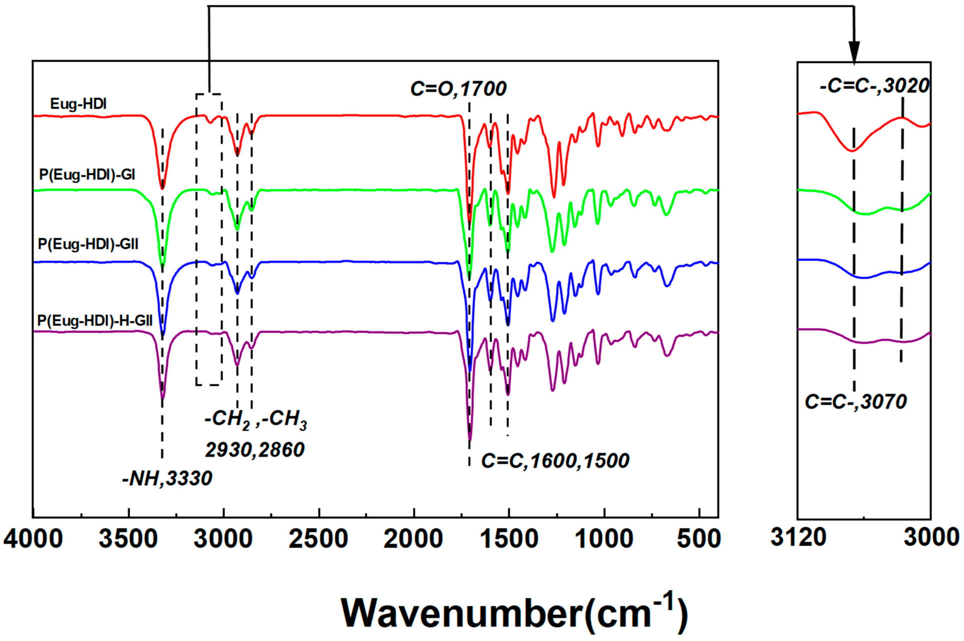

Figure S4. IR spectra of Eug-HDI and P(Eug-HDI) under different catalysts.

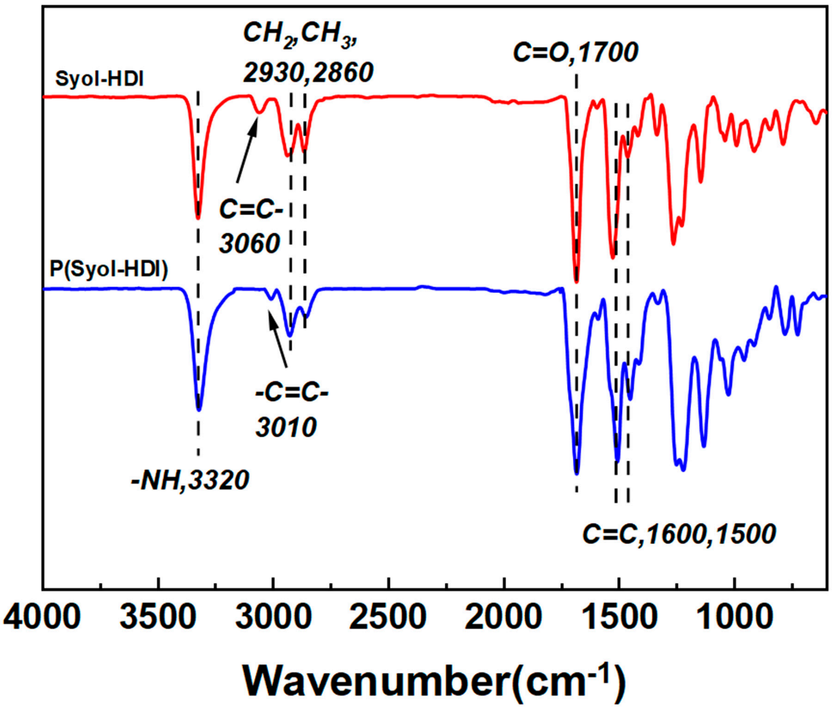

Figure S5. IR spectra of Syol-HDI and P(Syol -HDI).

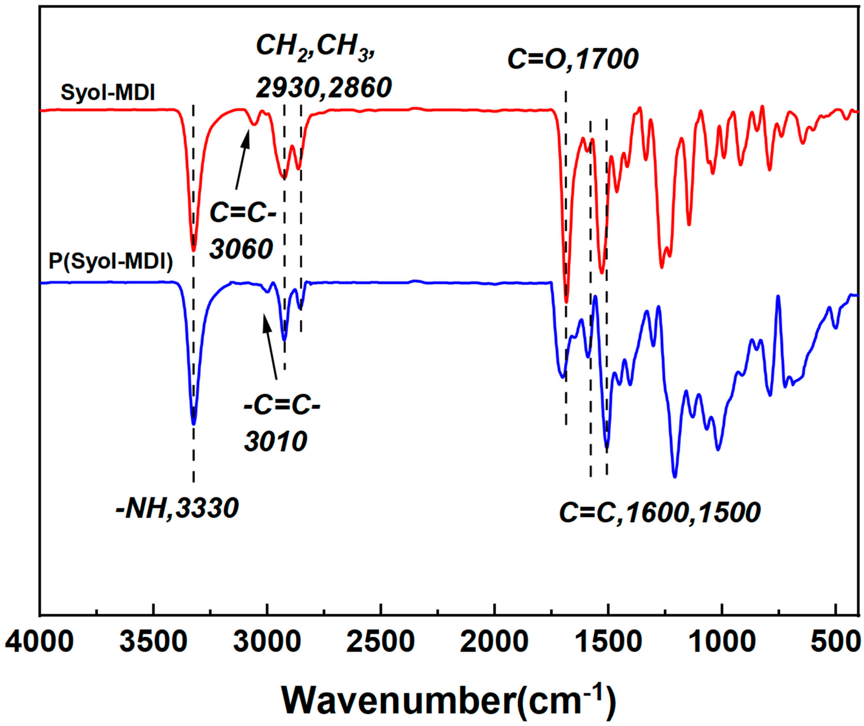

Figure S6. IR spectra of Syol-MDI and P(Syol - MDI).

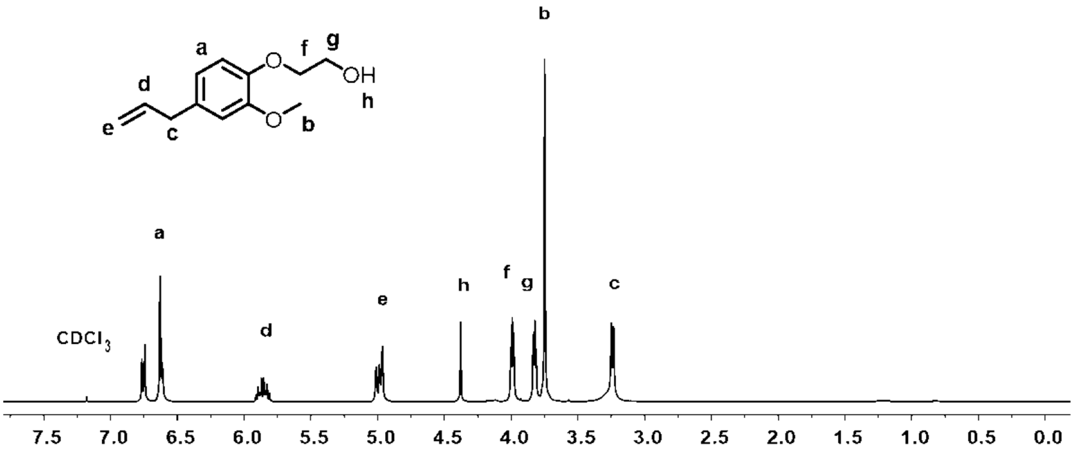

Figure S7. The ^{1}H NMR spectrum of Syol.

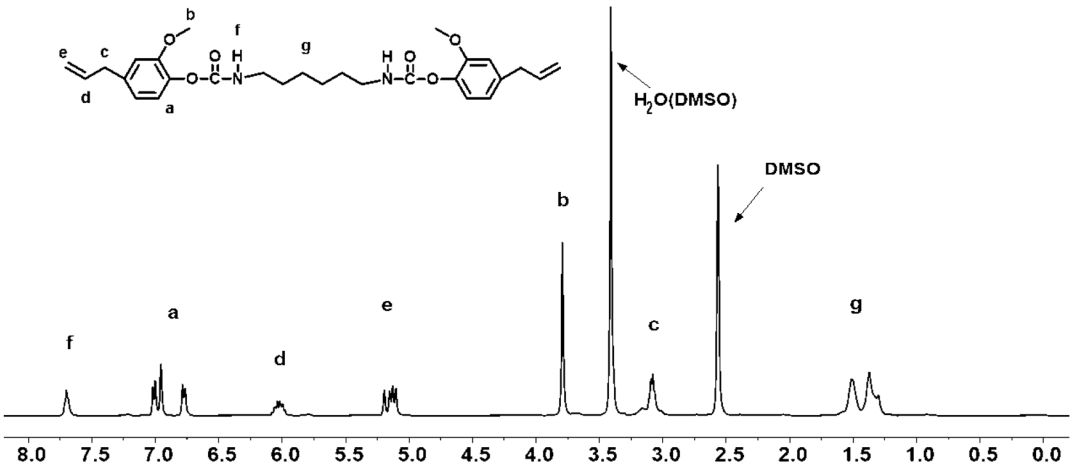

Figure S8. The ^{1}H NMR spectrum of Eug-HDI.

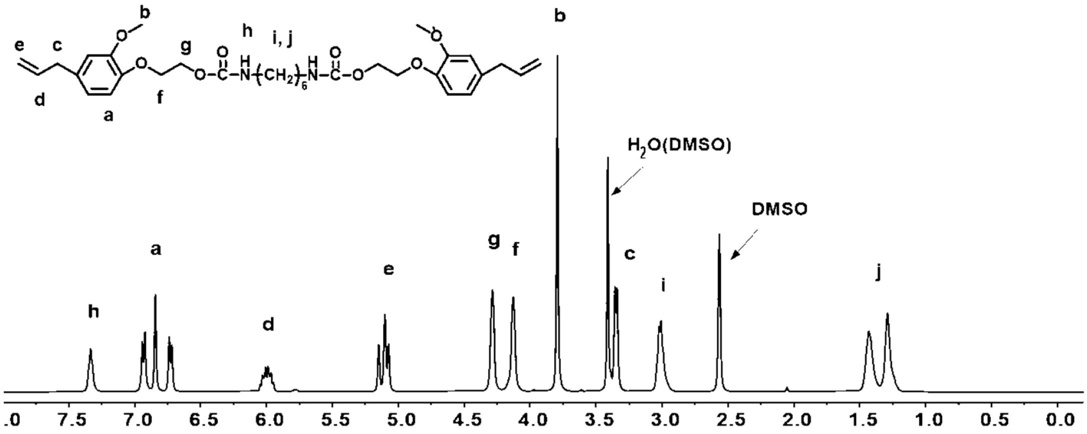

Figure S9. The ^{1}H NMR spectrum of Syol-HDI.

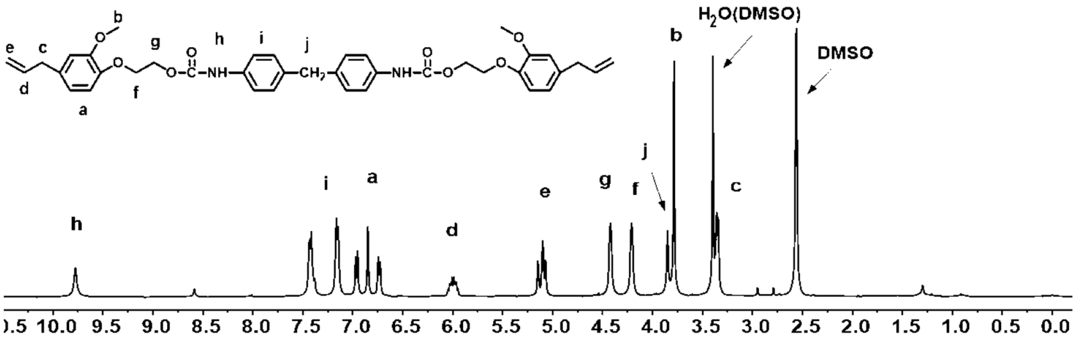

Figure S10. The ^{1}H NMR spectrum of Syol-MDI.

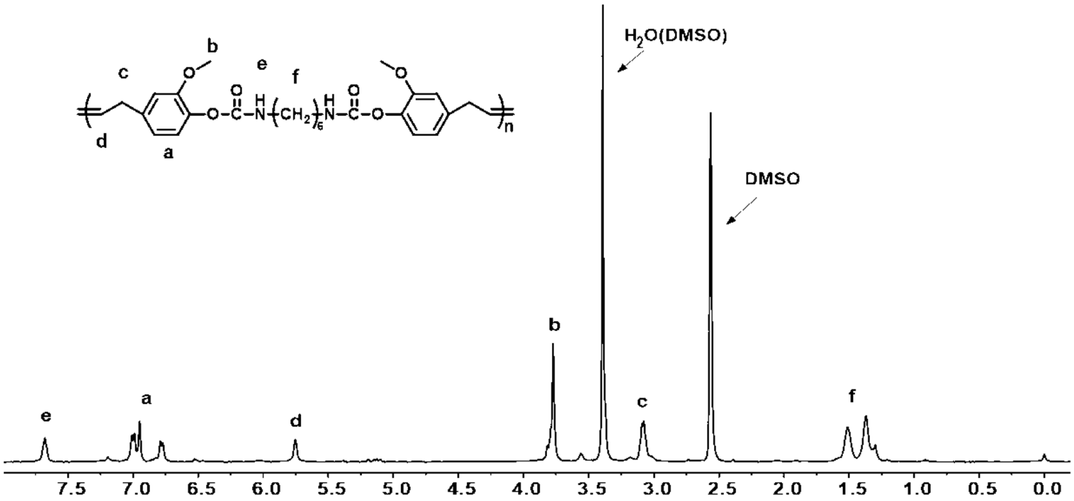

Figure S11. The ^{1}H NMR spectrum of P(Eug-HDI).

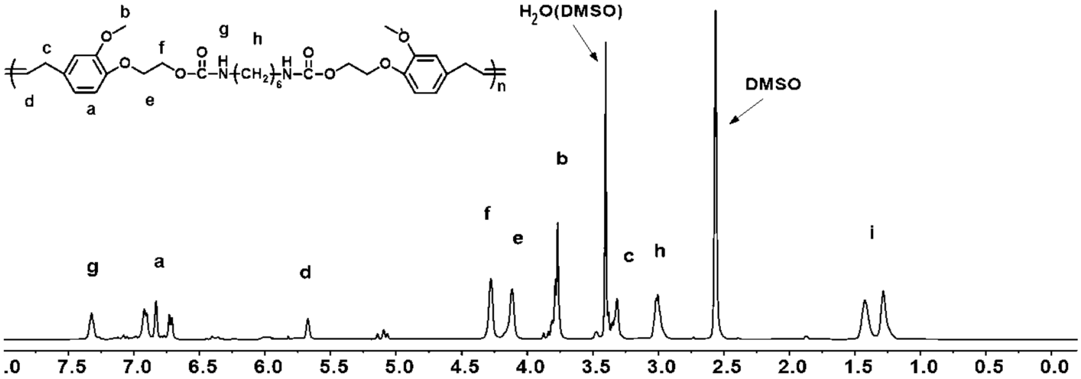

Figure S12. The ^{1}H NMR spectrum of P(Syol-HDI).

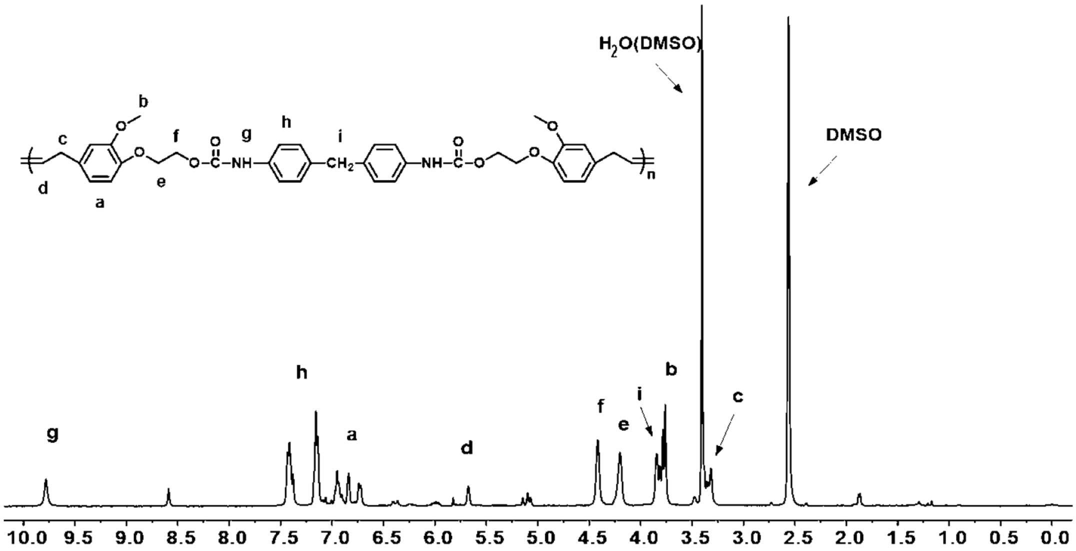

Figure S13. The ^{1}H NMR spectrum of P(Syol-MDI).

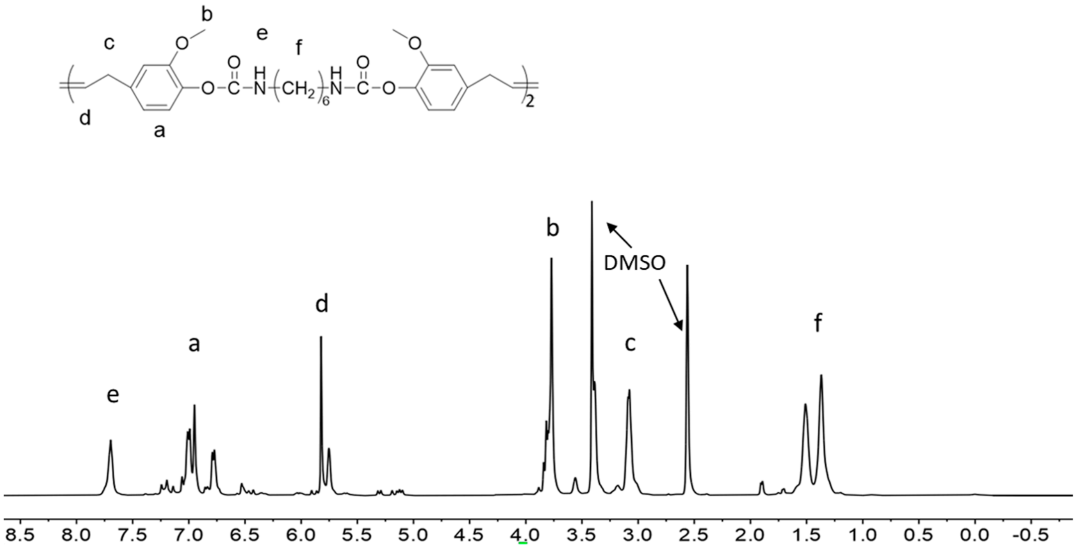

Figure S14. The ^{1}H NMR spectrum of product in methanol filtrateby dried by rotary evaporation.

Table S1. Olefin metathesis polymerization of Eug-HDI under different solvents, temperatures and reaction times.

**En-try**	**Eug-HDI/Cat. (molar ratio)**	**Solvent (8ml)**	**T (°C)**	**t (h)**	**Conv. (%)**	**M*n* （g/mol）**	***Đ***	***n***
1	100/1	THF	40	6	1.0	3000	1.40	6
2	100/1	THF	50	6	21.6	3100	1.36	6
3	100/1	THF	60	6	39.5	3300	1.33	7
4	100/1	THF	65	6	35.9	3400	1.44	7
5	100/1	TOL	40	6	10.0	2000	1.25	4
6	100/1	TOL	50	6	39.8	2000	1.28	4
7	100/1	TOL	60	6	63.5	2300	1.31	5
8	100/1	TOL	80	6	60.3	2600	1.53	6
9	100/1	TOL	100	6	57.6	2900	1.31	6
10	100/1	DCE	20	6	2.2	2000	1.21	4
11	100/1	DCE	30	6	29.4	2300	1.33	5
12	100/1	DCE	40	6	64.7	4400	1.41	9
13	100/1	DCE	50	6	60.5	4500	1.46	9
14	100/1	DCE	60	6	53.7	4700	1.44	10
15	100/1	DCE	80	6	41.9	5200	1.63	11
16	100/1	DCE	40	1.5	35.2	2700	1.46	5
17	100/1	DCE	40	3	37.4	3000	1.38	6
18	100/1	DCE	40	6	64.7	4400	1.41	9
19	100/1	DCE	40	9	64.7	3800	1.49	8
20	100/1	DCE	40	12	56.8	3500	1.52	7

Table S2. Olefin metathesis polymerization of Eug-HDI under different catalyst molar ratios.

Entry	Eug-HDI/Cat. (molar ratio)	Conv. (%)	M*n* （g/mol）	*Đ*	*n*
1	25/1	14.2	6400	1.26	13
2	50/1	34.3	5500	1.31	11
3	100/1	64.7	4400	1.41	9
4	200/1	44.2	2200	1.26	4
5	400/1	15.2	1600	1.77	3
6	500/1	0.5	1600	1.02	3

Table S3. Olefin metathesis polymerization of Eug-HDI with different metal salts.

Entry	Eug-HDI/Cat. (molar ratio)	Addition	Conv. (%)	M*n*[a] （g/mol）	*Đ*[b]	*n*[c]
1	100/1	-	64.7	4400	1.41	9
2	100/1	$MgCl_2$	37.4	2200	1.23	4
3	100/1	LiI	43.1	2200	1.27	4
4	100/1	$NiCl_2$	78.7	3800	1.52	8
5	100/1	$NiBr_2$	83.1	3000	1.39	6
6	100/1	NiI_2	86.6	2500	1.33	5
7	100/1	$CuCl_2$	-	-	-	-
8	100/1	CuCl	11.9	2200	1.24	4
9	100/1	CuBr	22.3	2100	1.22	4
10	100/1	CuI	49.9	2200	1.28	4
11	100/1	PPNCl	25.5	2100	1.21	4
12	100/1	PBQ	55.7	3300	1.34	7

a. Mn: number-average molecular weight. b. Dispersion index, used to describe the molecular weight distribution of polymer. c. n: degree of polymerization.
